# Effectors and effects of arginine methylation

**DOI:** 10.1042/BST20221147

**Published:** 2023-04-04

**Authors:** Yalong Wang, Mark T. Bedford

**Affiliations:** Department of Epigenetics and Molecular Carcinogenesis, The University of Texas MD Anderson Cancer Center, Houston, TX 77030, U.S.A.

**Keywords:** phase separation, PRMTs, R-loops, Tudor domains

## Abstract

Arginine methylation is a ubiquitous and relatively stable post-translational modification (PTM) that occurs in three types: monomethylarginine (MMA), asymmetric dimethylarginine (ADMA) and symmetric dimethylarginine (SDMA). Methylarginine marks are catalyzed by members of the protein arginine methyltransferases (PRMTs) family of enzymes. Substrates for arginine methylation are found in most cellular compartments, with RNA-binding proteins forming the majority of PRMT targets. Arginine methylation often occurs in intrinsically disordered regions of proteins, which impacts biological processes like protein–protein interactions and phase separation, to modulate gene transcription, mRNA splicing and signal transduction. With regards to protein–protein interactions, the major ‘readers’ of methylarginine marks are Tudor domain-containing proteins, although additional domain types and unique protein folds have also recently been identified as methylarginine readers. Here, we will assess the current ‘state-of-the-art' in the arginine methylation reader field. We will focus on the biological functions of the Tudor domain-containing methylarginine readers and address other domains and complexes that sense methylarginine marks.

## Introduction

Post-translational modifications (PTMs) increase the function diversity of the proteome by modifying a functional group of a protein. PTMs can occur at distinct amino acid side chains, and are usually mediated by enzymatic activity [1]. Protein methylation is a very common PTM type featuring the addition of methyl groups to certain amino acids including lysine, histidine and arginine. Of the 20 naturally occurring amino acids, arginine has the longest side chain and is positively charged, which makes it a good anchor for protein–protein and protein–nucleic acid interactions. Arginine methylation was first described by Paik and Kim over 50 years ago [2,3]. However, it was not until the methyltransferases that catalyze this modification were identified in yeast [4,5], that the functional analysis of arginine methylation really began. The methylation of arginine does not alter the net positive charge, but does change its shape/bulkiness and charge distribution, and thereby alters its hydrophobicity [6].

Arginine methylation is catalyzed by the family of protein arginine methyltransferases (PRMTs), which transfer a methyl group from S-adenosyl methionine (SAM) to the guanidino nitrogen of arginine, resulting in the formation of methylarginine and S-adenosylhomocysteine [7,8]. There are nine PRMT members in mammals which are further classified according to the type of methylation they are able to catalyze: all nine PRMTs are capable of monomethylation (MMA/Rme1), by adding a single methyl group on either terminal guanidinino nitrogen. Type I PRMTs (PRMT1, PRMT2, PRMT3, PRMT4/CARM1, PRMT6, PRMT8) catalyze the addition of a second methyl group to the same guanidinino nitrogen, generating asymmetric dimethylarginine (ADMA/Rme2a); Type II PRMTs (PRMT5, PRMT9) catalyze symmetric dimethylarginine (SDMA/Rme2s), by adding a second methyl group on the opposite terminal guanidinino nitrogen — one methyl group is placed on each of the two terminal guanidino nitrogen atoms; Type III PRMT (PRMT7) is limited to MMA [9]. PRMT1 is by far the most active PRMT which accounts for up to 85% of the ADMA activity in a cell [10,11], while PRMT5 is the primary Type II PRMT [12,13]. Importantly, a tenth Type II arginine methyltransferase exists, NDUFAF7, which is restricted to the mitochondria and is tightly complexed with its substrate, NDUFS2 [14,15]. Although NDUFAF7 harbors a seven-β-strand fold that is indicative of many methyltransferases, it is not closely related to the PRMTs. Between 0.5% and 4% of all arginine residues are methylated in mammalian cells, and the ratio of ADMA to MMA/SDMA is roughly 90 : 10, although this ratio can vary rather dramatically between cell types [16].

Most PRMTs methylate glycine/arginine-rich (GAR) motifs within their substrates [17], including PRMT1, 2, 3, 6 and 8. GAR motifs can be RGRG or RGGRGG repeats, and are often located in structurally disordered regions of proteins. The exceptions to this rule are CARM1, PRMT7 and PRMT9. The CARM1 methylation motif is very loosely defined, but usually harbors a proline residue, either C- or N-terminal to the arginine methylation site [18]. PRMT7 has a distinct substrate specificity for RXR motifs surrounded by basic residues [19]. To date, PRMT9 has only one known substrate called SF3B2, which it interacts with and modifies at a highly charged ..KRK.. motif. PRMT5 has been reported to prefer GAR motifs [20], while there is evidence that PRMT5 can also recognize a subset of CARM1 [21] and PRMT6 [22] methylation sites. PRMT6 can also methylate poly-R substrates that are devoid of any glycine residues [23]. A proteomic study from the Bonaldi group suggested that ∼30% of arginine methylation occurs at non-RG motifs [24]. So, although GAR motifs dominate as substrates, a substantial amount of arginine methylation occurs elsewhere.

An important insight in the field is that the different types of PRMTs such as PRMT1 and PRMT5 can share identical methylation sites on substrates [16]. As a consequence, depending on what PRMT is dominant (in a cell type or developmental stage), substrates may alter the prevalence of one type of methylarginine mark as opposed to another. Competing ADMA and SDMA marks could, therefore, allow tuning of biological processes [25–27]. Thus, through this process of ‘substrate scavenging', removal or inhibition of PRMT1 cause dramatic increases in global MMA and SDMA levels (concomitant with ADMA loss) [28], and loss of PRMT5 results in subtle increases in ADMA levels on certain substrates (concomitant with SDMA loss) [20,29]. Also, different PRMTs could also methylate the same substrates on distinct arginine sites. E2F1, for example, was methylated by PRMT1 at R109 site, while PRMT5 methylates E2F1 at R111 and R113 site [30].

Methylarginine marks are very stable, and in many cases, methylation is lost due to protein turnover rather than though an active enzymatic pathway. Arginine demethylases (RDMs) likely exist, and JMJD6 was identified as the first such candidate demethylase [31]. However, the assigned biological function of JMJD6 remains controversial [32], and it was initially misidentified as a phosphatidylserine receptor that plays a role in the apoptosis. JMJD6 is most likely a lysine hydroxylase [33], although it also has reported RNA demethylase activity [34] and tyrosine kinase activity [35]. To confuse matters further, JMJD6 and its close family members (JMJD5 and JMJD7), also possess protease activity which seems to be directed at arginine-methylated peptides [36,37]. Parenthetically, it has been shown that JmjC lysine demethylases can target artificially engineered methylarginine motifs. Specifically, KDM3A, which is a H3K9me2-specific demethylates can very efficiently demethylate a synthetic H3K9Rme1 peptide [38]. The same seminal study further reported that KDM4E and KDM5C both have RDM activity against naturally occurring methylation motifs on histone tails. KDM5C was also reported to demethylate ULK1 at R170 (R170me2s) [39]. Subsequently, the H3K9me2 demethylase, JMJD1B (KDM3B), was identified as H4R3me2s demethylase [40], but separating the functions of these dual lysine/arginine demethylases is difficult.

Like lysine methylation, arginine methylation of a substrate is often able to recruit an effector molecule to the newly created methyl-motifs. At about the same time (in 2001) that HP1 was discovered as the first reader of a methyllysine motif [41], SMN was identified as a binder of methylated arginine motifs [42,43]. SMN engages with arginine-methylated RNA-binding proteins through a Tudor domain. Subsequently, additional methylarginine effectors harboring Tudor domains were identified as well as a handful of non-Tudor domain effectors. Here, we will discuss the proteins and protein complexes that are recruited by arginine-methylated motifs to impact cellular process like transcription and splicing.

## Tudor domains as methylarginine readers

**Figure 1.**
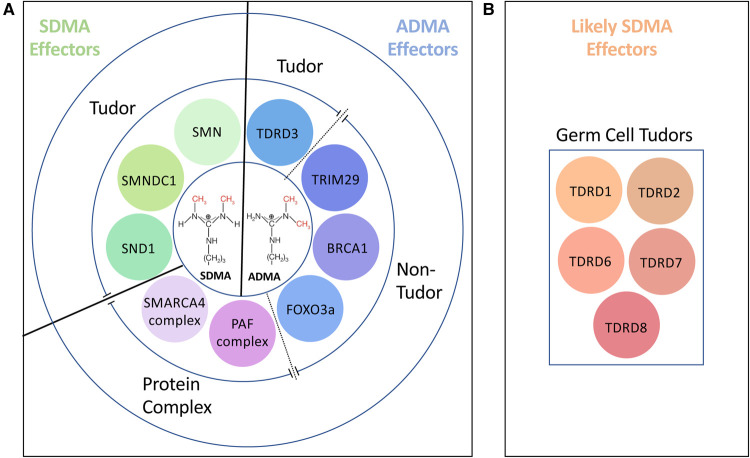
A Summary of the known and likely methylarginine effector proteins. (**A**) The central circle shows the structure of the SDMA and ADMA marks, which can be recognized by the listed protein and protein complexes. Concentric to that lie the known and well-characterized readers of methylarginine marks. Tudor domain-containing proteins that are SDMA effectors (green tones). ADMA effectors include a single Tudor domain protein, three proteins with unique folds (blue tones), and two protein complexes (purple tones). (**B**) A second group of likely SDMA effector are restricted to male germ cells and are all Tudor domain-containing proteins (orange tones).

Tudor domains are conserved protein folds originally identified in the Tudor protein encoded in *Drosophila melanogaster* [44]. Mutations of Tudor in the fruit fly are lethal to offspring, inspiring the name Tudor, as a reference to Tudor King Henry VIII from the English Tudor dynasty and the recurring fertility issues that his many wives (six in all) experienced. Tudor domains are typically about 60 amino acids in length and comprise four to five antiparallel β-strands to form a barrel-like structure. An essential component of the Tudor domain is the aromatic-binding cage structure formed by several aromatic amino acid residues. Structural studies showed that the aromatic cage is responsible for methylarginine and methyllysine recognition. There are roughly 30 Tudor domain-containing proteins in humans, but over 80 Tudor domains are identified, as many proteins have multiple copies of this domain. While methyllysine motifs can be read by at least eight different domain types (Tudor, Chromo, MBT, PHD, Ank, PWWP, WD40 and BAH), to date, Tudors are the only identified domain family to recognize methylarginine marks, and the details of these effectors will be addressed here (Figure 1). Importantly, the Tudor domains that read methyllysine motifs do not read methylarginine motifs, and *vice versa*.

*SMN* (survival motor neuron), is the disease gene for human spinal muscular atrophy (SMA), an autosomal disorder defined by progressive degeneration of lower motor neurons [45]. There are two highly homologous copies the gene, SMN1 and SMN2. The SMN1 and SMN2 genes are more than 99% identical, but SMN2 is aberrantly spliced to generate a sub-functional form of SMN called SMNΔ7. Mutations in both alleles of SMN1 cause SMA, and the levels of the protein produced by the remaining intact SMN2 gene modulate the severity of the phenotype in these patients. SMN acts as a molecular chaperone to regulate pre-mRNA splicing. It binds spliceosomal core proteins including SmD1, SmD3 and SmB/B’ through its Tudor domain, and this interaction is driven by symmetric methylation of the arginine residues in these splicing factors [42,43]. Importantly, mutations in the SMN1 Tudor domain are found in patients [46], and these mutations prevent binding to methylarginine motifs [47], highlighting the relevance of these interactions in disease. Structural studies confirmed that the SMN Tudor binds both SDMA and ADMA motifs, with lower affinity for ADMA [48]. The SMN Tudor domain forms a strongly bent antiparallel β-sheet, exhibiting a conserved negatively charged surface called an aromatic cage, which is shown to interact with GAR motifs of Sm proteins [49]. Compared with known ADMA binders, SMN displays a much wider binding groove near the aromatic cage, which might be a possible explanation why SMN is a very promiscuous effector molecule binding different motifs [50]. Apart from its role in splicing and snRNP assembly, SMN has many additional biological function including the regulation of both transcription and translation [51]. With regards to transcriptional regulation, it plays a role in termination of this process. Specifically, the SMN Tudor domain recognizes R1810me2s mark in the C-terminal region of RNA polymerase II (Pol II) and recruits senataxin, a component of the R-loop resolution machinery. The R1810me2s mark is catalyzed by PRMT5, and R1810A mutation (or depletion of PRMT5 or SMN) results in accumulated R-loops formation at the ACTB gene terminal region [52]. Recently, it was shown that the methlyarginine reader ability of Tudor domains, including that of SMN, regulates the formation of condensates *in vivo* [25]. In keeping with this observation, SMA-linked Tudor domain mutations prevent phase separation [53]. Elucidating the biological functions of SMN's methyl-reader ability will be facilitated now that a small molecule probe has been developed that selectively targets the Tudor domain of SMN [54].

*SMNDC1*, also referred to as SMNrp or SPF30, displays almost 50% identity with SMN [55]. SMNDC1 is reported as an essential splicing factor required for spliceosome maturation [56,57]. Peptide pulldown experiments showed that SMNDC1 Tudor binds SDMA motifs [47]. Structural studies show that like SMN Tudor, SMNDC1 Tudor domain also binds both SDMA and ADMA motifs, with weaker binding affinity for ADMA marks [48]. Compared with SMN Tudor, SMNDC1 displayed generally weaker methylarginine binding affinity [47,50]. Proteomic analysis of the SMNDC1 protein complex suggests roles for this effector not only in splicing but also the exosome RNA-decay pathway, ribosome biogenesis, snoRNA production [58] and chromatin remodelers [59].

*SND1*, short for Staphylococcal nuclease domain-containing protein 1, is also known as the p100, TSN or TDRD11, harbors four tandem SN domains at its’ N-terminus, followed by a C-terminal domain formed by a fusion of a Tudor domain and a truncated SN domain [60]. SND1 was initially identified as a transcriptional co-activator and has also been found as a component of RNA-induced silencing complex (RISC), which is involved in the RNA interference process. AGO2, a RISC component, is methylated by PRMT5, which generates a docking motif for SND1 and promote the degradation of AGO2-associated sRNAs [61]. The structures of human [62] and fly [63] SND1 Tudor domains have been solved, and in the case of the human study, the Tudor domain was complexed with methylarginine peptides from the PIWIL1 protein. SND1 Tudor domain preferentially to recognize SDMA marks, and like other arginine methylation readers, the binding of the SDMA marks by SND1 Tudor domain involves an aromatic cage. Moreover, the structure study reveals that the canonical Tudor domain of SND1 is not sufficient for binding the methylarginine peptides, as T688 and N823 from the SND1 SN domain are also involved in methylarginine binding. Protein domain microarray-based experiments identified the Tudor domain of SND1 as an effector of an SDMA motif in the E2F1 transcription factor [30]. Follow-up studies revealed that the recruitment of SND1 to arginine-methylated E2F1 results in altered splicing regulation of a subset of E2F1 transcriptional target genes [64]. Moreover, the SND1 Tudor directly interacts with arginine-methylated forms of many splicing factors including SmB/B’, SmD1, SmD3 and Sam68 [65,66].

*TDRD3* harbors one Tudor domain at its C-terminus, an OB-fold at the N-terminus and one UBA domain in between. The methylarginine binding ability of the TDRD3 Tudor domain was first identified by the Richard group using a peptide pulldown experiment [47], and independently confirmed by a protein microarray approach [67]. Further analysis showed that the Tudor domain of TDRD3 preferentially recognizes ADMA marks over SDMA and MMA marks [68], making it the only Tudor domain identified to-date that is an ADMA reader. Structural studies confirmed the affinity of TDRD3 for ADMA marks [50,69]. TDRD3 has the ability to recognize at least three histone methylation motifs (H4R3me2a, H3R17me2a and H3R2me2a) which are deposited by three different PRMTs (PRMT1, 4 and 6, respectively) [68]. Thus, it is not a dedicated effector of any one PRMT. TDRD3 is very tightly complexed with topoisomerase IIIB (TOP3B) [70,71] and it serves as a molecular bridge between TOP3B and arginine-methylated substrates [71]. Importantly, TOP3B can target both negative supercoiled regions of DNA and also double stranded RNA, where it can resolve RNA knots and catenanes [72]. Because the TDRD3/TOP3B complex can target both DNA and RNA, it will have distinct functions depending on the substrate it associates with. For example, the recruitment of TDRD3/TOP3B to promoters is important for resolving R-loops that form in the wake of RNA pol II [71], alternatively when the complex is associated with stress granules it likely targets tangled RNA molecules [73]. TDRD3 also interacts with non-histone PRMT substrates, including; (1) an ADMA mark on USP9X, to regulate USP9X deubiquitinating activity [74]; (2) the R1899 site on MED12 [75] recruits TDRD3/TOP3B to promote activator RNA interactions with the Mediator complex [76]; and (3) the Tudor domain of TDRD3 also ‘reads' the R1810me2a mark imbedded in the C-terminal domain of RNA Pol II [77], and may be a mechanism for recruit TOP3B activity to actively transcribed areas for R-loop resolution. As mentioned above, this R1810 site on tail of RNA Pol II is also targeted for SDMA modification by PRMT5 [52], which switches the reader of this site to SMN. Thus, the R1810 motif recruits either the TDRD3/TOP3B complex or the SMN/Senataxin complex, depending on if it is decorated with an ADMA or SDMA mark, and these two complexes resolve R-loops that occur at the transcriptional promoter or termination site, respectively.

## Tudor domain that might be methylarginine effectors

TDRD3 and SMN are the best studies Tudor domain-containing effectors of methylarginine marks. A sub-group of TDRD proteins are localized exclusively in the male germ cells and are found associated with germinal granules (nuage structures), which are critical for germline development [78]. However, they can be aberrantly expressed in a cancer setting [79]. Fly PIWI proteins and their mouse orthologs (MIWI proteins) are heavily arginine-methylated testis-specific proteins that are recognized by these Tudor domain proteins. The germline-specific Tudors are not well-characterized as readers of methylarginine marks, but evidence does exist that they are effectors.

*TDRD1* has four Tudor domains, and it interacts with PIWIL2, a Piwi-like RNA-binding protein involved in piRNA metabolic process. PIWIL2 is arginine methylated at its N-terminus, and a SDMA peptide from this region can pulldown TDRD1 from cell lysates [80], indicating TDRD1 Tudors recognize SDMA marks. Furthermore, an R-to-K mutant of PIWIL2 disrupts its interaction with TDRD1, and arginine methylation inhibitor treatment abolishes the interaction between PIWIL2 and TDRD1, further indicating arginine methylation as a binding determinant [81]. It is not clear which of the four Tudor domains harbor SDMA reader activity, but the 3rd and 4th Tudor domains of TDRD1, in isolation, can be recruited to phase-separated foci [25], suggesting that these domains might be active SDMA readers.

*TDRD2* is also named as TDRKH, and it is single Tudor domain has been reported to interact with arginine-rich N-terminus of PIWIL1 and PIWIL4 [62], and the interaction is decreased when PRMT5 is silenced [82]. A structure study of TDRD2 Tudor in complex with PIWIL1 peptide reveals that the Tudor domain of TDRD2 preferentially recognizes the unmethylated arginine-rich sequence from PIWIL1 [83,84]. More work is needed to resolve these inconsistencies.

*TDRD6* is a Tudor domain-only containing protein with eight Tudor domains. TDRD6 interacts with PIWIL1, and an SDMA-modified peptide (but not an ADMA-modified peptide) from PIWIL1 could pulldown TDRD6 [85]. Since TDRD6 has eight Tudor domains, further study is required to characterize which of the eight Tudor domains has the SDMA-binding properties, although the 5th Tudor domain of TDRD6, can be recruited to phase-separated foci [25].

*TDRD7* also participates in spermiogenesis [86] and is co-immunoprecipitated with PIWIL2 [81], but it is not clear if the interaction is arginine methylation dependent, and if Tudor domain is required for the interaction.

*TDRD8* (STK31) is a testis-specific protein that binds PIWI; however, the methyl-dependent nature of this interaction was never tested [87]. The 3rd Tudor domain of TDRD8, can be recruited to phase-separated foci [25].

## Arginine methylation readers that do not harbor a Tudor domain

Although Tudors are currently the only known domain type with multiple members that bind methylarginine marks, other protein folds and complexes have demonstrated an affinity for this PTM type. Interestingly, all these non-canonical effectors are ADMA readers, and most recognize non-GAR motif methylation deposited by CARM1.

*PAF1c*, the PAF1 complex, composed of five proteins (Paf1, Ctr9, Cdc73, Rtf1 and Leo1), accompanies RNA Pol II during transcriptional elongation to regulate a large and growing list of processes. A peptide pulldown experiment shows that H3R17me2a peptide could selectively pulldown all five PAF1c subunits [88]. PAF1c binds to histone H3 tails harboring H3R17me2a mark on CARM1-methylated histone octamers. Either CARM1 knockdown or CARM1 enzyme-deficient mutant knock-in results in decreased H3K17me2a, accompanied by the reduction in PAF1c recruitment. These results suggest that PAF1c acts as an arginine methylation effector for H3R17me2a. None of the PAF1c subunits has Tudor domains, and it would be interesting to establish which subunit is the H3R17me2a effector.

*BRCA1* binding to p300 is stabilized by arginine methylation. CARM1 methylates the p300 acetyltransferase at many arginine residues to regulate its activity towards histones. The R754 position within the KIX domain of p300 is one of these sites. The KIX domain is known to interact with many proteins and a candidate approach was taken here to identify BRCA1 as a methyl-sensitive effector [89]. Peptide pulldown experiments using methylated R754 peptides reveal that BRCT domain of BRCA1 preferentially interact with the ADMA peptide. The ADMA binding properties of the BRCA1–BRCT domain are likely unique, as an expanded screen of additional BRCT domains did not yield any methylarginine effectors.

*SMARCA4*, also known as Brg1, is an ATPase subunit of SWI/SNF chromatin-remodeling complex. SMARCA4 was identified as an effector for PRMT1-mediated H4R3me2a mark using a comparative peptide (H4R3me2a vs H4R3me2s) pulldown approach from nuclear extracts, followed by mass spectrometry identification of the interacting proteins [90]. The authors mapped the region of interaction to the HELICc domain of SMARCA4. Using an isothermal titration calorimetry assay they validated direct binding between the HELICc domain-containing region of SMARCA4 and H4R3me2a peptide, but not H4R3me0 or H4R3me2s peptides.

*FOXO3a* was identified as a methyl-dependent reader of the chromatin-remodeling factor Pontin, which is methylated by CARM1 [91]. ChIP-seq was performed for methylated Pontin, and then motif analysis identify transcription factors binding sites that are enriched under these peaks, FOXO3a being one such factor. Treatment with CARM1-specific inhibitors markedly decreases the interaction between Pontin and FOXO3a, and Pontin RK and RA mutants fail to bind FOXO3a. Analysis of a set of truncated FOXO3a proteins found that methylated pontin directly bind the CR3 domain of FOXO3a. A putative aromatic cage was identified in the CR3 domain and mutation of two aromatic residues in this region blocked effector binding.

*TRIM29* belongs to the RING-less group of TRIM family proteins [92]. TRIM29 was recently identified as an effector protein for methylated NFIB [93]. NFIB is methylated by CARM1 at R388. GFP-trap experiments were performed to identify proteins that would interact with GFP-NFIB-R388 but not with the R388K mutant. TRIM29 was shown to interact with NFIB in a CARM1-dependent manner using CARM1 inhibitor and CARM1 KO cell lines. Pulldowns experiments show that NFIBme2a peptide but not NFIBme0 control peptide directly interacts with a recombinant form of the C-terminal domain of TRIM29. An AlphaFold analysis of the predicted structure of this C-terminal region identified a putative aromatic cage for methyl-reading. Importantly, TRIM29 is not only an effector for the CARM1-deposited methyl-mark on NFIB, but also interacts selectively with an ADMA fibrillin GAR peptide, indicating that TRIM29 might be a rather general ADMA reader.

*WDR5* is a WD-40 repeat-containing protein that interacts with the N-terminal tail of histone H3. It is a component of many different protein complexes that are recruited to chromatin. Its interaction with H3 has been reported to be inhibited by the H3R2me2a mark [94–96], and promoted by the H3R2me2s mark [97]. Recent studies have validated the ability of the H3R2me2a modification to block WDR5 recruitment, but revealed that the H3R2me0, -me1 and -me2s states are equally permissive for this interaction [98].

## Concluding remarks

Here, we summarized the field of methylarginine effector proteins, which includes Tudor and non-Tudor proteins. The most recent effectors to be identified (SMARCA4, FOXO3a and TRIM29) do not harbor Tudor domains. This indicated that the screens for Tudor domain binders of methylarginine motifs may now be saturated. Furthermore, these findings may reflect the advances in mass spectrometry sensitivity, which allows for many more candidates to be identified in peptide pulldown and GFP-trap experiments. This increase in the screening depth is needed to identify subtle complex component changes due to the recruitment of methylarginine readers. These types of experiments are often ‘noisier’ than usual because the highly charged nature of GAR motifs, perhaps explaining why these mass spectrometry-based proteomic approaches have worked better for the identification of effectors of CARM1-generated motifs, which generally do not harbor more than one arginine residue.

## Perspectives

- Given the abundance of arginine methylation modifications, and the paucity of known readers, it is expected that there will be additional methylarginine effector proteins. Tudor domains-containing proteins have been thoroughly screened as methyl effectors. The most recently identified methylarginine effectors are either proteins with novel folds or protein complexes.
- Arginine methylation readers likely all form aromatic cages for methylarginine mark recognition. New protein structure prediction programs such as AlphaFold will help identify potential effector protein with aromatic cages that are similar to know effectors.
- It is possible that methylarginine reader cages could form as a result of two distant parts of a protein being brought together through folding. This concept may also be true for protein complexes, where the interacting surfaces of two proteins could fold into a functional aromatic cage, and thus jointly contribute aromatic residues for the formation of the cage. Also, further structure studies will help us better understand the mechanism surrounding the selective recognition of SDMA and ADMA motif.

## Abbreviations

ADMA: asymmetric dimethylarginine
CARM1: co-activator associated methyltransferase
GAR: glycine/arginine rich
KDM: lysine demethylase
MMA: monomethylation
polymerase II: Pol II
PRMT: protein arginine methyltransferase
PTM: post-translational modification
RISC: RNA-induced silencing complex
SAM: S-adenosyl methionine
SDMA: symmetric dimethylarginine
SMA: spinal muscular atrophy
TOP3B: topoisomerase IIIB
